# Exploratory Analysis of the Correlations Between Physiological and Biomechanical Variables and Performance in the CrossFit^®^ Fran Benchmark Workout

**DOI:** 10.3390/jfmk10040387

**Published:** 2025-10-05

**Authors:** Alexandra Malheiro, Pedro Forte, David Rodríguez Rosell, Diogo L. Marques, Mário C. Marques

**Affiliations:** 1 Department of Sport Sciences, University of Beira Interior, 6201-001 Covilhã, Portugal; alexandra.malheiro@ubi.pt (A.M.); diogoluis.sequeira@gmail.com (D.L.M.); mariomarques@mariomarques.com (M.C.M.); 2 Research Center in Sports Sciences, Health Sciences, and Human Development (CIDESD), 6201-001 Covilhã, Portugal; 3 Department of Sports, Instituto Politécnico de Bragança, 5301-854 Bragança, Portugal; 4 Department of Sports, Higher Institute of Educational Sciences of the Douro, 4560-708 Penafiel, Portugal; 5 Research Center for Active Living and Wellbeing (LiveWell), Instituto Politécnico de Bragança, 5301-854 Bragança, Portugal; 6 Physical Performance and Sports Research Center, Universidad Pablo de Olavide, 41013 Seville, Spain; drodros@upo.es; 7 Department of Sport and Informatics, Universidad Pablo de Olavide, 41013 Seville, Spain

**Keywords:** CrossFit, maximal strength, aerobic fitness, training experience, sex differences, exploratory analysis

## Abstract

**Background**: The multifactorial nature of CrossFit performance remains incompletely understood, particularly regarding sex- and experience-related physiological and biomechanical factors. **Methods**: Fifteen trained athletes (8 males, 7 females) completed assessments of anthropometry, estimated one-repetition maximums (bench press, back squat, deadlift), squat jump (SJ), maximal oxygen uptake (VO_2_max), ventilatory responses ($\dot{V}$E), and heart rate (HR). Spearman, Pearson, and partial correlations were calculated with Holm and false discovery rate (FDR) corrections. **Results**: Males displayed greater body mass, lean and muscle mass, maximal strength, and aerobic capacity than females (all Holm-adjusted *p* < 0.01). Experienced athletes completed Fran faster than beginners despite broadly similar anthropometric and aerobic profiles. In the pooled sample, WOD time showed moderate negative relationships with estimated 1RM back squat (ρ = −0.54), deadlift (ρ = −0.56), and bench press (ρ = −0.65) before correction; none remained significant after Holm/FDR adjustment, and partial correlations controlling for training years were further attenuated. **Conclusions**: This exploratory study provides preliminary evidence suggesting that maximal strength may contribute to Fran performance, whereas conventional aerobic measures were less influential. However, given the very small sample (n = 15, 8 males and 7 females) and the fact that no relationships remained statistically significant after correction for multiple testing, the results must be regarded as preliminary, hypothesis-generating evidence only, requiring confirmation in larger and adequately powered studies.

## 1. Introduction

CrossFit is a high-intensity sport that combines weightlifting, gymnastics, and metabolic conditioning, requiring athletes to perform complex movements under fatigue with efficiency and control [1]. Performance is influenced by biomechanical, physiological, and psychological factors, including strength, aerobic capacity, neuromuscular coordination, and mental resilience [2,3,4]. Athletes with greater muscular fitness and aerobic efficiency generally show superior outcomes, particularly in workouts demanding sustained effort and rapid transitions [3,4,5]. Among these attributes, cardiorespiratory fitness (e.g., VO_2_max and ventilatory thresholds) is associated with quicker recovery between bouts [6], while muscular strength enables athletes to lift heavier loads and maintain technical execution under fatigue [7]. Optimal CrossFit performance, therefore, appears to require a combination of physical capacity and psychological readiness [8,9,10,11].

Strength and aerobic capacity have consistently been highlighted as foundational attributes [12,13]. Strength, particularly maximal values derived from the back squat (BS), deadlift (DL), and bench press (BP), supports Olympic lifting, stability under load, and bodyweight efficiency [3,14]. Aerobic capacity, typically assessed Via VO_2_max and ventilatory thresholds, sustains work output and facilitates recovery in longer-duration workouts [12,15,16]. These qualities are commonly evaluated through 1RM estimations, jump protocols, and treadmill gas exchange [7,17,18]. In recent years, velocity-based methods using the load–velocity (L–V) relationship have provided a valid, efficient, and lower-risk alternative to direct 1RM testing [19,20,21,22]. The application of strength, aerobic, and neuromuscular tests in CrossFit research provides a multifaceted physiological and biomechanical profile relevant for monitoring adaptation and predicting performance [23,24,25,26,27]. Yet, the literature does not fully agree on which predictors dominate. Some studies emphasize strength as the key determinant [14], while others highlight aerobic capacity, particularly among female athletes [28,29]. Mangine et al. [4] reported that strength and anaerobic power were most predictive for men, whereas Martínez-Gómez et al. [10] found aerobic factors to be stronger predictors in women. These findings indicate that sex and training experience may modulate the relative contribution of different physical qualities, but the extent and consistency of these effects remain unclear.

To examine such relationships in an ecologically valid setting, benchmark workouts such as Fran are frequently employed. Performance in Fran, expressed as total completion time, reflects the athlete’s ability to integrate force production, movement efficiency, and endurance capacity under high-intensity conditions [3]. This workout is one of the most widely recognized CrossFit benchmarks, consisting of descending sets of thrusters and pull-ups, and is valued for its simplicity, repeatability, and ability to simultaneously tax strength, aerobic fitness, and coordination [9,29,30]. Its ecological validity is further reinforced by the standardized load prescriptions and scaling practices that preserve intensity across different fitness levels [31,32]. Although Fran provides a relevant and applied context, it represents only a single CrossFit workout. Previous studies have examined predictors of CrossFit performance, but they have typically addressed isolated domains (e.g., strength or aerobic capacity) or broader batteries of tests, without simultaneously integrating biomechanical, physiological, and anthropometric correlates within the same applied workout [4,12,14,28,29]. Moreover, few studies have explicitly accounted for sex-related differences and training experience as potential modifiers of these relationships. Given the limited availability of integrated, multifactorial analyses in applied CrossFit settings—and the inherent challenges of recruiting large, homogeneous samples—there remains a gap in understanding how these combined attributes contribute to benchmark performance under ecologically valid conditions.

Therefore, the present investigation was designed as an exploratory, hypothesis-generating study aimed at examining potential biomechanical and physiological correlates of performance in the benchmark workout Fran, with sex treated as a grouping factor and training experience incorporated as a covariate to account for potential confounding. We hypothesized that biomechanical and physiological variables would differ by sex, be affected by expertise, and display relationships with performance outcomes.

## 2. Materials and Methods

### 2.1. Study Design

A cross-sectional exploratory design was used to examine potential associations between physiological, neuromuscular, and biomechanical variables and functional performance in CrossFit athletes. The primary outcome variable was the time to complete the benchmark WOD “Fran”, while the independent variables included strength, cardiorespiratory, and movement-based variables. Fifteen trained CrossFit athletes (8 males, 7 females) participated in the study and were evaluated at a single time point using a set of standardized laboratory and field-based assessments [33]. Training experience (years of CrossFit practice) was recorded as a continuous variable and later used as a covariate in statistical models, rather than as a categorical grouping factor. This approach minimized subgroup instability and maintained statistical power in a small sample.

**Figure 1:**
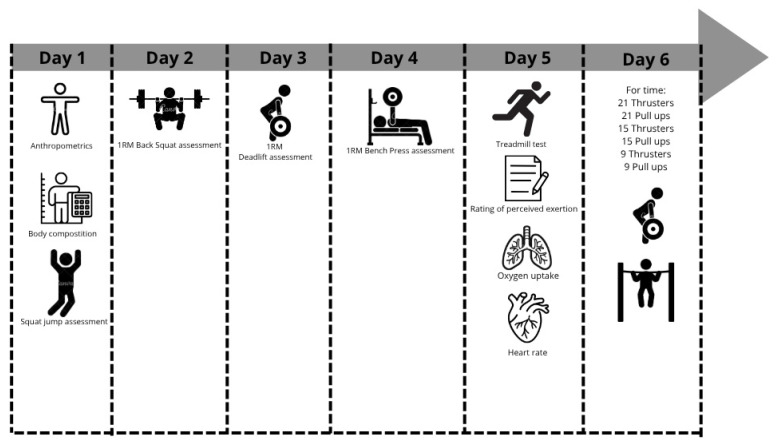
Schematic representation of the experimental protocol.

The study was conducted over six non-consecutive days (with at least 48 h of rest in between sessions), during which participants underwent comprehensive assessments of body composition, strength variables (1RM in BS, DL, and BP, and squat jump evaluation), and cardiorespiratory fitness (VO_2max,_ heart rate (HR), and resting perceived exertion (RPE)). On day 6, participants completed the WOD “Fran”, a workout consisting of 21-15-9 repetitions of thrusters and pull-ups, performed for time, which served as the primary performance outcome. The experimental protocol was structured into three sequential phases to allow for comprehensive profiling of physiological and biomechanical capacities relevant to high-intensity functional training (Figure 1). The first phase included baseline assessments, consisting of anthropometrics, body composition analysis, a squat jump test [17], and strength testing using incremental 1RM protocols for the BS [23,24], BP [23], and DL [17]. The second phase focused on the cardiorespiratory evaluation, which included a treadmill-based test to determine VO_2max_, heart rate response [18], and rating perceived of exertion (RPE) [34]. The final phase, on day 6, involved execution of the “Fran” WOD under controlled conditions. During the WOD, internal load was continuously monitored to quantify physiological stress and assess real-world functional capacity. All assessments were performed under standardized and controlled laboratory or field conditions, following established protocols to ensure consistency, reliability, and reproducibility across all measurement domains.

Although 20 athletes volunteered, only 15 completed all phases (8 males: 27.9 ± 5.8 y, 75.2 ± 5.0 kg, 176.5 ± 5.7 cm; 7 females: 27.4 ± 3.1 y, 61.0 ± 4.5 kg, 165.0 ± 3.4 cm). Given the small overall sample and the exploratory nature of the investigation, no a priori power analysis was performed; instead, effect sizes and confidence intervals are reported to inform the design of future confirmatory studies.

No a priori sample size calculation was conducted, as this study was explicitly designed as exploratory and hypothesis-generating, based on the convenience sample of trained CrossFit athletes available during the recruitment period. Effect sizes and confidence intervals were, therefore, prioritized in the analysis to provide preliminary evidence to guide future confirmatory research.

### 2.2. Sample

A group of 20 trained CrossFit athletes (10 men and 10 women) volunteered to participate in this study. However, two males and three females were absent from at least one testing session (due to illness, injury, or unjustified reasons). Thus, the final sample consisted of 15 participants, comprising 8 males (age: 27.9 ± 5.8 years; body weight: 75.2 ± 5.0 kg; height: 176.5 ± 5.7 cm) and 7 females (age: 27.4 ± 3.1 years; body weight: 61.0 ± 4.5 kg; height: 165.0 ± 3.4 cm). All participants were actively training in CrossFit at the time of data collection, with a minimum of one year of continuous practice and a weekly training frequency of at least three sessions. Training history (years of CrossFit practice) was recorded for each participant and later used as a continuous covariate in statistical analysis to account for its potential confounding influence, rather than to define discrete subgroups. Weekly training load and competition level are acknowledged as potential confounders. All participants were informed of the study procedures and provided written informed consent. The study was conducted under the Declaration of Helsinki and approved by the institutional ethics committee (CE-UBI-Pj-2024-090-ID2772).

All participants completed a familiarization session one week before testing, including practice trials for the treadmill protocol, L–V testing, and jump assessments. This ensured technical competence and minimized learning effects. Test–retest reliability was established in a subset of participants (n = 5), yielding ICC values ranging from 0.88 to 0.94 for strength measures and 0.86–0.92 for jump variables, consistent with previous reports [35].

### 2.3. Maximum Strength Assessment in the Back Squat, Bench Press, and Deadlift

Recent strength testing research supports the high reliability of load–velocity (L–V) based strength estimation. For example, González-Badillo & Sánchez-Medina [36] demonstrated a very tight association between mean propulsive velocity (MPV) and %1RM in bench press, which remained stable across sessions. Similarly, Martínez-Valência et al. [37] reported intraclass correlation coefficients (ICC) > 0.99 for velocity measurement devices across a wide range of loads, indicating that such devices can reliably capture concentric barbell velocity in strength tasks. In addition, device reliability for exercises such as the back squat and bench press has been confirmed, e.g., Lawson et al. [38] found good to excellent ICCs across these lifts, which supports the methodological decision in this study to use velocity-based estimations and ensure consistency in testing.

Prior to each physical test, participants completed a structured warm-up to ensure safety, readiness, and consistent neuromuscular performance. A general warm-up (10 min of light aerobic activity such as cycling or jogging, followed by dynamic mobility drills and muscle activation exercises) was conducted before each test. This was followed by test-specific warm-up routines, described in detail below. Participants received verbal encouragement to ensure maximal effort in all repetitions.

All strength tests were conducted using a free-weight Olympic barbell (20 kg, Eleiko, Halmstad, Sweden) and monitored with a Chronojump linear position transducer (Boscosystem, Barcelona, Spain; 3 m cable, 160 Hz sampling rate, 24-bit ADC), which was attached to the bar to record mean concentric velocity. Each test employed a progressive loading protocol, concluding near 80% of the participants’ one-repetition maximum (1RM) [19]. This approach minimized fatigue and injury risk while maintaining ecological validity, estimating the 1RM value.

#### 2.3.1. Back Squat

Back squat performance was assessed using a submaximal, velocity-based loading protocol. A detailed description of the testing procedure used in this study has been reported elsewhere [23,24]. For the execution of each repetition, the participants placed the bar on the upper trapezius. In addition, participants were asked to always perform the eccentric phase at a controlled velocity, while the concentric phase was always performed at maximal intended velocity. The specific warm-up included dynamic mobility drills, and 2 sets of 8 and 6 repetitions, respectively, with 20 kg (3 min apart).

Following warm-up, testing began at 20 kg for all participants, with progressive increments of 10–20 kg depending on bar velocity, until the movement velocity was less than 0.70 m·s^−1^ (~80% 1RM). At each load, participants completed 2–3 repetitions with maximal concentric intent, interspersed with 2–3 min of rest. Only technically valid repetitions performed with full range of motion (i.e., thighs below parallel) were analyzed. The fastest repetition per load, as determined by the highest mean velocity of the propulsive phase (MPV) [26], was used for velocity profiling. Estimated 1RM (1RMest) was calculated for each individual from the MPV attained against the heaviest load (kg) lifted in the progressive loading test, as follows: (100 × load)/(25.961 × MPV^2^) − (50.71 × MPV) + 117 [24].

#### 2.3.2. Bench Press

A detailed description of the testing procedure used in this study has been reported elsewhere [25]. Participants lay supine on a flat bench using a self-selected grip. Specific warm-up consisted of Dynamic shoulder and thoracic mobility drills (e.g., scapular push-ups, shoulder circles), and 2 sets of 8 and 6 repetitions, respectively, with 20 kg (3 min apart).

Testing began with 20 kg and progressed in 5–10 kg increments until the movement velocity was less than 0.50 m·s^−1^ (~80% 1RM). At each load, participants completed 2–3 repetitions with maximal concentric velocity. Rest intervals were 2–3 min. Proper technique (i.e., feet flat, bar lowered under control to the chest without bounce, and no excessive lumbar extension) was required in each repetition. The fastest technically valid repetition per load was retained for the subsequent analysis. Estimated 1RM was calculated for each individual from the MPV attained against the heaviest load (kg) lifted in the progressive loading test, as follows: (100 × load)/(8.4326 × MPV^2^) − (73.501 × MPV) + 112.33 [36].

#### 2.3.3. Deadlift

Deadlift assessment was based on the protocol by Benavides-Ubric et al. [17], using a conventional stance and pronated grip. The specific warm-up included: joint mobility (e.g., hip circles, spinal flexion/extension) and 2 sets of 8 and 6 deadlift repetitions with 0.3 kg and 20 kg loads, respectively.

The initial load was set at 20 kg for all participants. Then, the progressive loading sequence was structured as follows: (i) 20 kg increments until mean propulsive velocity (MPV) < 0.80 m·s^−1^ (3 reps per load); (ii) 10 kg increments for MPV 0.80–0.60 m·s^−1^ (2 reps per load).

Testing concluded when the MPV values corresponded approximately to 80% of the 1RM (0.58 m·s^−1^). Technical validity required a vertical bar path, full hip and knee extension, and no rebending of the knees. The fastest valid repetition per load was used for the subsequent analysis. Rest intervals of 3 min were maintained between sets. Estimated 1RM was calculated for each individual from the MPV attained against the heaviest load (kg) lifted in the progressive loading test, as follows: (100 × load)/(−71,681 × MPV) + 121,118 [17].

### 2.4. Squat-Jump Test

In this test, participants began from a stationary semi-squat position on a portable force platform (42 × 59 cm, Chronojump Boscosystem, Barcelona, Spain). With knees flexed to approximately 90 degrees, participants performed a maximal vertical jump without countermovement, extending their legs in a single explosive action. Arms remained on the hips throughout, and landing was standardized on the toes to ensure consistency. Performance was assessed based on vertical jump height (cm), which was calculated from flight time using Chronojump’s integrated software (sampling frequency: 1000 Hz) (VO_2_ Master Cloud platform v0.99.1, Vernon, Canada). Each participant performed 3 attempts with 50–60 s recovery time. The highest jump was retained for analysis.

### 2.5. Treadmill Test for Respiratory Gas Exchange and Heart Rate Evaluation

Participants performed a graded treadmill test to assess maximal oxygen uptake (VO_2max_) and estimate energy expenditure under standardized conditions. Respiratory gas exchange was measured using a breath-by-breath portable gas analyzer (VO2 Master Health Sensors Inc., Vernon, BC, Canada), and heart rate (HR) was continuously monitored with a chest strap device (Polar S610, Kempele, Finland). The gas analyzer was calibrated for both volume and gas concentrations prior to each session, following the manufacturer’s guidelines to ensure accurate and reliable measurements. The VO2 Master system, as well as the chest strap device, has been validated in previous research [39,40], demonstrating strong agreement with criterion laboratory-based systems, making it suitable for field and applied sport science settings. VO_2_max was determined using standard criteria (achievement of volitional exhaustion plus a plateau in VO_2_ despite increasing speed, RER ≥ 1.10, or HR ≥ 90% age-predicted maximum). Ventilatory thresholds (VT1, VT2) were determined using the V-slope method and confirmed by ventilatory equivalents (VE/VO_2_, VE/VCO_2_) and excess CO_2_ criteria. Threshold identification was conducted by two experienced evaluators blinded to the participant subgroup. Inter-rater reliability for VT determination was excellent (ICC = 0.91).

To minimize learning effects, all participants completed a familiarization session one week before data collection, including practice trials for the treadmill, L–V strength assessment, and jump tests. Reliability testing was conducted in a subset of 5 participants, yielding intraclass correlation coefficients (ICCs) between 0.86 and 0.94 across strength and jump measures, consistent with previous reports in similar populations.

After a 3 min warm-up at 8–8.5 km·h^−1^, the test began at 9 km·h^−1^ with a fixed incline of 1%. This starting speed was based on performance during prior familiarization sessions. The protocol involved incremental increases in treadmill speed by 0.3 km·h^−1^ every 25 s, continuing until volitional exhaustion. Participants were verbally encouraged to maintain their effort for as long as possible. VO_2_ and HR were recorded breath by breath and averaged over 30 s intervals for analysis, allowing for precise determination of VO_2max_ and mean physiological values throughout the test.

### 2.6. Workout of the Day (WOD)

The benchmark CrossFit workout “Fran” is a widely recognized and frequently used protocol within high-intensity functional training (HIFT), valued for its simplicity and capacity to challenge multiple fitness components such as strength, endurance, and coordination [30]. Comprising descending sets of 21, 15, and 9 repetitions of thrusters and kipping pull-ups, “Fran” is designed to be completed in the shortest possible time, generating a potent cardiovascular and muscular stimulus. Its structure makes it especially effective for assessing exercise tolerance and overall fitness capacity in functional training settings [9]. Participants completed Fran according to their individual training level, using either the Rx or scaled prescription. For correlation analyses, all results were based on athletes’ actual completion times. We acknowledge that scaling introduces heterogeneity and may influence comparability; this was considered in the interpretation of the results.

In the present study, participants completed either Rx (as prescribed) version, with a barbell load of 43 kg for males and 29 kg for females, or a scaled version adjusted to individual abilities. Scaled options included lighter loads and pull-up modifications (e.g., band assistance or jumping pull-ups), ensuring inclusivity without compromising the workout’s intensity [32]. This individualized approach aligns with best practices in HIFT programming, where scaling is used to preserve safety and ecological validity across varying fitness levels [7]. Emphasis on proper execution, such as achieving full squat depth and elbow lockout in thrusters, reflects the importance of standardized performance metrics for both safety and reliable data collection [41]. While “Fran” has also been explored in broader contexts, such as its psychosocial benefits [42], its utility in evaluating physical performance under high-intensity conditions remains clear [31].

### 2.7. Rating of Perceived Exertion (RPE)

The RPE was used to assess participants’ subjective perception of effort following two key phases of the protocol: the incremental treadmill test and the CrossFit benchmark workout (i.e., Fran WOD). RPE was recorded using the modified Borg CR10 scale, which ranged from 0 (no effort) to 10 (maximum exertion). Participants were instructed on how to interpret and use the scale prior to testing. After completing each effort (treadmill test and Fran WOD), participants were asked to report their overall perceived exertion based on the full-body experience of fatigue, breathlessness, and muscular strain. This measure provided valuable insight into internal load and exertional stress, complementing physiological data such as heart rate and oxygen uptake [43].

### 2.8. Variables Extracted for Analysis

From each physical and physiological test, a specific set of variables was extracted. For the BS, BP, and DL (performed at approximately 80% of 1RM), the following metrics were recorded: estimated one-repetition maximum (1RM, in kilograms), mean concentric velocity (m/s), mean power output (watts), and mean force output (newtons). In the squat jump (SJ), variables included jump height (cm), calculated from flight time, take-off velocity (m/s), mean power output (watts), and time of flight (seconds). The cardiorespiratory test provided mean VO_2_ and maximal VO_2_ (both in mL·kg^−1^·min^−1^), as well as mean heart rate (beats per minute). Anthropometric and body composition assessments yielded body mass (kg), height (cm), lean mass (kg), muscle mass (kg), and fat mass percentage (%). Additionally, age (years) was recorded as a demographic variable. All these variables were included in the correlation analysis with WOD time performance (in seconds), first across the total sample and subsequently stratified by sex (male vs. female) or training experience (beginners vs. experts).

### 2.9. Statistical Analysis

Standard statistical methods were used for descriptive calculations (mean ± SD or median [IQR] as appropriate). Normality was examined with the Shapiro–Wilk test and homogeneity of variances with Levene’s test. Because several variables deviated from normality and the sample was small, non-parametric and estimation-based approaches were prioritized. Sex differences were examined with independent-samples *t*-tests or Mann–Whitney U tests as appropriate, with effect sizes (Hedges g or Cliff’s δ) and 95% confidence intervals (CI) considered the primary outcomes.

Associations between Fran performance time and physiological or biomechanical variables were assessed using Pearson and Spearman correlations, and partial correlations controlling for training experience (years) to account for potential confounding. Correlation coefficients (r, ρ) and their magnitude were emphasized, while *p*-values were reported only descriptively.

To limit the risk of Type I error from multiple testing, Holm-Bonferroni and Benjamini–Hochberg false discovery rate (FDR) corrections were applied [44]. Both unadjusted and adjusted *p*-values are provided for transparency, but the results were interpreted primarily in terms of effect size magnitude and precision, consistent with the exploratory purpose of the study.

All analyses were performed in IBM SPSS Statistics (Version 23; IBM Corp., Armonk, NY, USA) and JASP (Version 0.18.3.0; JASP Team, Amsterdam, The Netherlands). Statistical significance was interpreted cautiously, emphasizing effect size magnitude and confidence intervals in keeping with the study’s exploratory, hypothesis-generating purpose.

## 3. Results

### 3.1. Exploratory Analysis of Sex Differences in Anthropometric, Physiological, and Biomechanical Characteristics

Table 1 summarizes the descriptive characteristics of the participants by sex, including mean ± SD, Hedges g effect sizes, and 95% confidence intervals. Men were taller and heavier and exhibited greater lean and muscle mass compared with women, with large effect sizes (e.g., body mass g ≈ 2.1, height g ≈ 1.7, lean mass g ≈ 2.0). Strength-related variables (estimated 1RM back squat, deadlift, and bench press) likewise showed large sex differences (g > 1.5). In contrast, fat mass percentage displayed small and non-meaningful differences (g ≈ −0.5). Aerobic fitness variables (VO_2_max and ventilatory thresholds) and neuromuscular power (squat jump height) showed small-to-moderate sex differences, with confidence intervals overlapping zero. Training experience, recorded as years of continuous CrossFit practice, was entered as a covariate and, therefore, not treated as a grouping factor in these analyses.

Mann–Whitney U tests revealed several sex-related differences. However, given the small sample, we emphasize the effect sizes (Hedges g) and their confidence intervals as the most informative outcomes. Large effect sizes were observed for body mass, height, lean mass, muscle mass, and estimated strength values, while aerobic and neuromuscular variables showed smaller or overlapping effects. These results should, therefore, be interpreted in terms of magnitude and direction rather than statistical significance alone. Compared with females, males displayed significantly greater body mass (U = 55.0; *p* = 0.0006; Hedges g = 2.13 [95% CI: 0.86, 3.40]), height (U = 53.5; *p* = 0.0022; g = 1.72 [0.53, 2.91]), lean mass (U = 56.0; *p* = 0.0003; g = 2.07 [0.82, 3.33]) and muscle mass (U = 56.0; *p* = 0.0003; g = 2.02 [0.79, 3.26]). Absolute strength values were also higher in males, with greater estimated 1RM in the back squat (U = 54.0; *p* = 0.0012; g = 1.80 [0.64, 2.97]), deadlift (U = 52.0; *p* = 0.0037; g = 1.65 [0.51, 2.78]) and bench press (U = 56.0; *p* = 0.0003; g = 1.95 [0.76, 3.14]). In addition, males demonstrated moderately higher squat-jump height and VO_2_max, though effect sizes were smaller and confidence intervals overlapped zero. Training experience (years of CrossFit practice) was entered as a covariate in subsequent analyses and was not used to form subgroups.

### 3.2. Association Between Key Variables and WOD Performance

In Table 2, males presented significantly higher values than females in several key variables. Body mass was 79.0 ± 8.8 kg in males versus 62.3 ± 5.3 kg in females (U = 55.0; *p* = 0.006), and height was 179 ± 9 cm versus 166 ± 5 cm (U = 53.5; *p* = 0.010). Lean mass and muscle mass were also greater in males (65.0 ± 8.1 kg and 33.9 ± 4.5 kg, respectively) than in females (48.0 ± 4.2 kg and 22.6 ± 2.8 kg; U = 56.0; *p* = 0.005 for both). Maximal strength showed similar sex effects: estimated 1RM back squat 124 ± 28 kg vs. 84 ± 13 kg (U = 54.0; *p* = 0.009), deadlift 151 ± 28 kg vs. 92 ± 15 kg (U = 52.0; *p* = 0.015), and bench press 83 ± 16 kg vs. 43 ± 7 kg (U = 56.0; *p* = 0.005). Cardiorespiratory variables also differed, with VO_2_max 55.1 ± 5.8 vs. 43.2 ± 6.7 mL·kg^−1^·min^−1^ (U = 55.0; *p* = 0.006), mean VO_2_ 42.3 ± 6.2 vs. 31.5 ± 5.3 mL·kg^−1^·min^−1^ (U = 53.0; *p* = 0.010), and $\dot{V}$Emax 157 ± 28 vs. 107 ± 18 L·min^−1^ (U = 56.0; *p* = 0.005). All differences remained significant after Holm correction. No other anthropometric or physiological variables differed significantly, and inclusion of years of CrossFit practice as a covariate did not materially change these findings.

Table 3 shows that WOD completion time was inversely associated with maximal strength variables. Estimated 1RM back squat correlated at r = −0.57 (*p* = 0.026), deadlift at r = −0.55 (*p* = 0.033), and bench press at r = −0.61 (*p* = 0.016). Similar relationships were obtained with Spearman coefficients (ρ = −0.54 to −0.65). Fat mass and age displayed weaker, non-significant correlations (r ≈ −0.24 to 0.28; *p* > 0.30). Partial correlations adjusted for years of CrossFit practice remained negative for the main strength variables (r ≈ −0.30 to −0.40) but did not reach significance. After applying Holm and false discovery rate (FDR) corrections for multiple testing, no correlation remained statistically significant.

Following the correlation analysis summarized in Table 3, maximal strength variables showed moderate inverse relationships with WOD completion time (Pearson r ranging from −0.55 to −0.61; Spearman ρ ≈ −0.54 to −0.65). Fat mass and age displayed weak and non-meaningful relationships (r ≈ −0.24 to 0.28), and all other variables were trivial in magnitude. Partial correlations adjusted for years of CrossFit experience remained negative for the main strength measures (r ≈ −0.30 to −0.40), though of smaller magnitude. Partial correlations adjusted for years of CrossFit experience remained negative for the main strength measures (r ≈ −0.30 to −0.40), though smaller in magnitude. However, these analyses were clearly underpowered given the small sample size and, therefore, should be regarded as descriptive and exploratory only. These relationships were moderate in effect size but did not remain statistically significant after correction for multiple testing (Holm or FDR). Accordingly, the results should be interpreted in terms of the magnitude and direction of associations rather than statistical significance alone, reinforcing their exploratory and hypothesis-generating nature.

**Figure 2:**
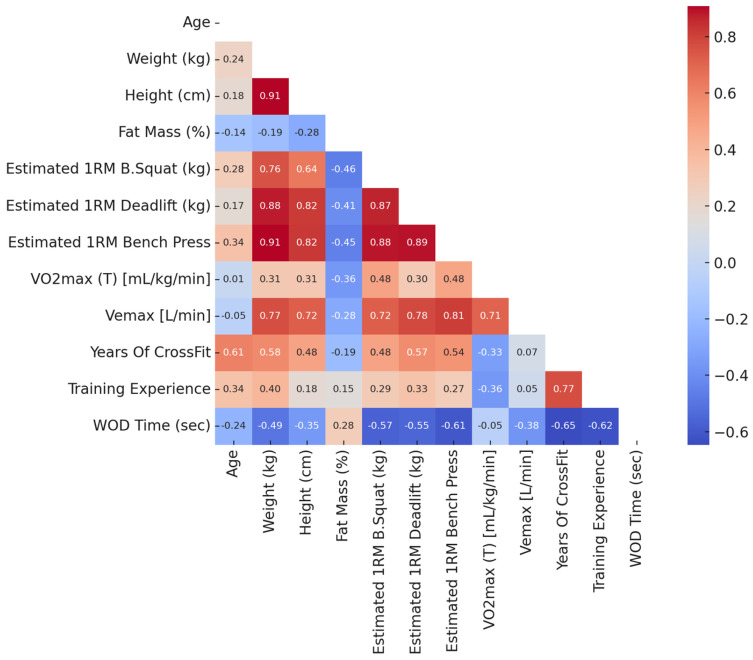
Pearson correlation heatmap among anthropometric, strength, aerobic, experience, and performance variables (n = 15). Stronger negative values (blue) indicate faster Fran completion times, whereas positive values (red) reflect slower performance. Only the lower triangle is shown for clarity. Although several raw correlations with WOD time appeared moderate in magnitude, none remained statistically significant after Holm–Bonferroni or FDR correction for multiple testing.

Figure 2 provides a visual representation of these relationships, highlighting stronger negative relationships between WOD completion time and maximal strength variables for Spearman correlation test. However, it is important to note that this should be analyzed with caution due to the loss of significance in Holm and FDR corrections.

## 4. Discussion

This study aimed to explore physiological and biomechanical correlates of performance in the benchmark CrossFit workout Fran, with attention to potential sex- and experience-related effects. We hypothesized that (i) male athletes would display superior strength, jump performance, and aerobic capacity, (ii) more experienced athletes would complete the WOD faster, and (iii) these physiological characteristics would correlate with performance in a subgroup-specific manner. The findings partly supported these hypotheses; however, it is important to note that all analyses must be interpreted as exploratory and hypothesis-generating due to the small sample size and the loss of statistical significance after correction for multiple testing.

Consistent with established literature on neuromuscular and morphological dimorphism [45,46,47,48], males demonstrated significantly higher body mass (79.0 ± 8.8 kg vs. 62.3 ± 5.3 kg), stature (179 ± 9 cm vs. 166 ± 5 cm), lean mass (65.0 ± 8.1 kg vs. 48.0 ± 4.2 kg), and muscle mass (33.9 ± 4.5 kg vs. 22.6 ± 2.8 kg) compared with females. They also outperformed females in estimated one-repetition maximum (1RM) for the back squat, deadlift, and bench press (all *p* < 0.01 after Holm correction), and in cardiorespiratory variables such as VO_2_max (55.1 ± 5.8 vs. 43.2 ± 6.7 mL·kg^−1^·min^−1^) and maximal ventilation ($\dot{V}$Emax: 157 ± 28 vs. 107 ± 18 L·min^−1^). These sex-specific differences align with previous work in functional fitness and strength sports [7,49]. Nevertheless, the small subgroups (n = 8 and n = 7) yield wide confidence intervals and prevent confirmatory inference.

Athletes with ≥4 years of CrossFit practice completed Fran faster than those with less experience, supporting the relevance of accumulated training history, technical proficiency, and pacing strategies described in earlier reports [11,16]. However, one year of CrossFit experience was treated as a covariate instead of a grouping factor; no additional physiological predictors emerged. This finding reinforces the idea that practice-related adaptations, skill acquisition, movement economy, and tactical execution may mediate performance more than isolated physiological traits [4,12].

Correlation analyses across the total sample revealed moderate negative relationships between WOD completion time and maximal strength variables—estimated 1RM back squat (r = −0.57, *p* = 0.026), deadlift (r = −0.55, *p* = 0.033), and bench press (r = −0.61, *p* = 0.016)—with similar Spearman coefficients (ρ = −0.54 to −0.65). These relationships are in line with previous findings identifying maximal strength as a primary determinant of functional fitness performance [3,14,29,50]. Fat mass and age showed only weak, non-significant correlations. Crucially, none of these associations survived Holm or FDR correction, reinforcing their preliminary and descriptive character. Partial correlations controlling for training experience were smaller in magnitude and underpowered, and, therefore, should be interpreted as exploratory only.

Within these constraints, the present findings generate hypotheses for programming. They tentatively suggest that maximal strength in fundamental lifts (back squat, deadlift, bench press) may contribute meaningfully to benchmark WOD performance, whereas traditional cardiorespiratory markers such as VO_2_max may play a lesser role in this specific workout. However, the current evidence is inconclusive, and no practical prescriptions can be drawn from these results. Instead, these exploratory observations highlight the need for replication in larger, adequately powered studies that incorporate multiple benchmark workouts, competition-level contexts, and broader assessments, including psychological, technical, and tactical determinants of performance.

### 4.1. Limitations

Several limitations must be underscored. First, the sample size was very small (n = 15; 8 males and 7 females). This severely restricts statistical power, increases the risk of spurious or inflated correlations, and explains why none of the initially significant relationships survived multiple-comparison correction. As a result, the present findings must be interpreted strictly as preliminary, hypothesis-generating evidence rather than confirmatory outcomes [41,51].

In addition, the partial correlation analyses that controlled for training experience were not adequately powered, given the small sample size. Although they provided useful exploratory insights, these results should be interpreted with extreme caution and cannot be generalized. Larger samples are required to properly evaluate the independent contribution of training experience to performance outcomes. Multiple comparisons further heighten the risk of Type I error, and even with effect-size reporting, our findings remain preliminary. The analysis was restricted to a single WOD (Fran), limiting generalizability to other CrossFit workouts with different physiological demands [2,32].

Another limitation is the ecological scope of the design. While Fran is a widely recognized CrossFit benchmark, it represents only a single workout format. As such, the present findings may not generalise to other CrossFit workouts with different physiological or technical demands, nor to formal competition contexts where pacing strategies, judging standards, and psychological stressors also influence performance. Additionally, although both Rx and scaled versions of Fran were accepted to preserve ecological validity and inclusivity, this introduces heterogeneity that may confound direct comparisons.

Psychological, technical, and cognitive determinants of performance-known contributors to success in CrossFit, were not assessed [8,51]. Finally, although VO_2_max was determined using established criteria (volitional exhaustion with VO_2_ plateau, RER ≥ 1.10, or HR ≥ 90% of predicted maximum), not all participants may have fulfilled every criterion simultaneously, so these values are best interpreted as reflecting near-maximal aerobic capacity.

In summary, this study suggested that maximal strength in fundamental lifts could possibly explain the performance in the CrossFit Fran workout, whereas conventional aerobic measures appear less influential. However, these findings are inconclusive and should only be viewed as exploratory, requiring replication in larger, longitudinal cohorts that incorporate multiple WODs, formal competition-level classification, and broader assessments of physiological and technical determinants of performance.

### 4.2. Practical Aplications

Despite these limitations, preliminary trends suggest that maximal strength in fundamental lifts (back squat, deadlift, bench press) may contribute more to Fran performance than conventional aerobic markers. For practitioners, this implies that monitoring and developing strength qualities remain relevant for performance in short, high-intensity CrossFit benchmarks. In practical terms, coaches may consider systematically tracking athletes’ strength alongside conditioning variables to better understand their readiness and performance potential. However, these insights must be interpreted with caution and should not yet be used to prescribe training strategies; they remain exploratory hypotheses that require confirmation in larger, adequately powered studies incorporating multiple workouts and broader performance determinants.

## 5. Conclusions

This study provided exploratory, hypothesis-generating results suggesting that males displayed higher values in anthropometric, biomechanical, and physiological parameters compared with females. Maximal strength variables, particularly the back squat, deadlift, and bench press, showed moderate negative relationships with performance in the benchmark CrossFit workout Fran, whereas conventional aerobic markers (e.g., VO_2_max, ventilatory indices) appeared less influential in this specific task. However, given the very small sample (n = 15) and the fact that none of these relationships remained statistically significant after correction for multiple testing, the findings should be regarded as preliminary and inconclusive. Future research with larger and adequately powered cohorts, multiple benchmark workouts, and broader assessment of psychological and technical determinants is required to confirm or refute these exploratory observations.

## Figures and Tables

**Table 1:** Sex comparisons are presented in Table 1 with effect sizes and 95% confidence intervals.

Variable	Male Mean ± SD	Female Mean ± SD	Hedges g (95% CI)
Age (years)	28.9 ± 6.9	27.3 ± 5.6	0.24 (−0.78, 1.25)
Weight (kg)	79.8 ± 9.8	61.9 ± 4.8	2.13 (0.86, 3.40)
Height (cm)	178.5 ± 9.4	164.9 ± 4.1	1.72 (0.53, 2.91)
Fat Mass (%)	17.7 ± 5.0	19.7 ± 2.2	−0.49 (−1.52, 0.54)
Lean Mass (kg)	65.7 ± 9.5	49.6 ± 3.3	2.07 (0.82, 3.33)
Muscle Mass (kg)	33.9 ± 2.9	22.1 ± 1.6	4.61 (2.67, 6.54)
Years of CrossFit	4.4 ± 4.2	3.7 ± 1.5	0.19 (−0.83, 1.21)
VO_2max_ (mL/kg/min)	57.4 ± 6.7	44.4 ± 5.1	2.04 (0.79, 3.30)
1RM Back Squat (kg)	124.1 ± 24.2	82.4 ± 10.5	2.03 (0.80, 3.31)
1RM Deadlift (kg)	155.4 ± 27.9	111.7 ± 6.3	1.97 (0.73, 3.20)
1RM Bench Press (kg)	89.0 ± 17.7	47.1 ± 7.9	2.81 (1.38, 4.23)
SJ height (cm)	32.2 ± 5.6	26.7 ± 5.2	0.95 (−0.12, 2.02)
WOD Time (sec)	374.9 ± 153.6	476.1 ± 189.9	−0.56 (−1.59, 0.48)

Note: Values are mean ± SD unless otherwise indicated. Between-sex differences tested using independent-samples *t*-tests or Mann–Whitney U as appropriate, with effect sizes (Hedges g or Cliff’s δ) reported. Holm–Bonferroni adjusted *p*-values were applied to control for multiple testing. Only strength and anthropometric variables remained statistically significant after correction; no other differences survived.

**Table 2:** Between-sex differences in key anthropometric, strength, and cardiorespiratory variables in trained CrossFit athletes (n = 15). Values are presented as mean ± standard deviation. Group comparisons were performed using the Mann–Whitney U test, with *p*-values adjusted for multiple testing using the Holm procedure. Only variables showing significant differences or high physiological/biomechanical relevance are reported. VO_2_max: maximal oxygen uptake; $\dot{V}$Emax: maximal minute ventilation.

Variables	Males (n = 8) Mean ± SD	Female (n = 7) Mean ± SD	Mann–Whitney U	*p* (Holm adj.)
Body mass (kg)	79.0 ± 8.8	62.3 ± 5.3	55.0	0.006
Height (cm)	179 ± 9	166 ± 5	53.5	0.010
Lean mass (kg)	65.0 ± 8.1	48.0 ± 4.2	56.0	0.005
Muscle mass (kg)	33.9 ± 4.5	22.6 ± 2.8	56.0	0.005
Estimated 1RM Back Squat (kg)	124 ± 28	84 ± 13	54.0	0.009
Estimated 1RM Deadlift (kg)	151 ± 28	92 ± 15	52.0	0.015
Estimated 1RM Bench Press (kg)	83 ± 16	43 ± 7	56.0	0.005
VO_2_max (mL·kg^−1^·min^−1^)	55.1 ± 5.8	43.2 ± 6.7	55.0	0.006
Mean VO_2_ (mL·kg^−1^·min^−1^)	42.3 ± 6.2	31.5 ± 5.3	53.0	0.010
Max Ventilation $\dot{V}$Emax (L·min^−1^)	157 ± 28	107 ± 18	56.0	0.005

Note: Only variables showing significant differences or high physiological/biomechanical relevance are presented. No other measured variables differed significantly between sexes.

**Table 3:** Bivariate and partial correlations between *Fran* workout completion time and key anthropometric, strength, and body-composition variables in trained CrossFit athletes (n = 15). Values are Pearson’s correlation coefficient (r) and Spearman’s rank correlation coefficient (ρ) with corresponding *p*-values; partial r represents Pearson correlations adjusted for years of CrossFit practice. Holm and false discovery rate (FDR) procedures were applied to adjust for multiple comparisons. Negative coefficients indicate that higher values of the predictor variable are associated with faster completion times (better performance).

Variable	Pearson r (*p*)	Spearman ρ (*p*)	Partial r adj. Years of CF (*p*)	*p* Holm adj	*p* FDR adj
Age	−0.24 (0.394)	−0.19 (0.502)	0.26 (0.342)	0.394	0.394
Fat Mass (%)	0.28 (0.315)	0.23 (0.403)	0.21 (0.462)	0.946	0.349
Estimated 1RM Back Squat (kg)	−0.57 (0.026)	−0.54 (0.039)	−0.39 (0.155)	0.318	0.111
Estimated 1RM Deadlift (kg)	−0.55 (0.033)	−0.56 (0.028)	−0.30 (0.284)	0.359	0.111
Estimated 1RM Bench Press	−0.61 (0.016)	−0.65 (0.009)	−0.40 (0.137)	0.225	0.111

Note: Pearson, Spearman, and partial correlations between Fran completion time and selected variables. Reported values are correlation coefficients with corresponding *p*-values in parentheses. Holm and FDR adjusted *p*-values account for multiple testing. Although some raw *p*-values were < 0.05, no relationships remained statistically significant after correction.

## Data Availability

The raw data supporting the conclusions of this article will be made available by the authors on request.

## Abbreviations

The following abbreviations are used in this manuscript:

1RM	One-repetition maximum
BS	Back squat
BP	Bench press
DL	Deadlift
SJ	Squat jump
HR	Heart rate
HRV	Heart rate variability
VO_2_max	Maximal oxygen uptake
VO_2_mean	Mean oxygen uptake
VE	Ventilation
RPE	Rating of perceived exertion
WOD	Workout of the Day
HIFT	High-intensity functional training
Rx	As prescribed (standard workout load in CrossFit)
